# Bis(trimethyl­phenyl­ammonium) tetra­bromidobis(4-chloro­phen­yl)stannate(IV)

**DOI:** 10.1107/S160053680904940X

**Published:** 2009-11-25

**Authors:** Kong Mun Lo, Seik Weng Ng

**Affiliations:** a Department of Chemistry, University of Malaya, 50603 Kuala Lumpur, Malaysia

## Abstract

The Sn^IV^ atom in the title salt, [N(CH_3_)_3_(C_6_H_5_)]_2_[SnBr_4_(C_6_H_4_Cl)_2_], exists in a distorted all-*trans* SnC_2_Br_4_ octa­hedral geometry. The Sn^IV^ atom lies on a center of inversion. Weak inter­molecular C—H⋯Br hydrogen bonding is observed between trimethyl­phenyl­ammonium cations and the Sn complex anion in the crystal structure.

## Related literature

For bis­(4-dimethyl­amino­pyridinium) tetra­bromidodiphenyl­stannate, see: Yap *et al.* (2008 ▶).
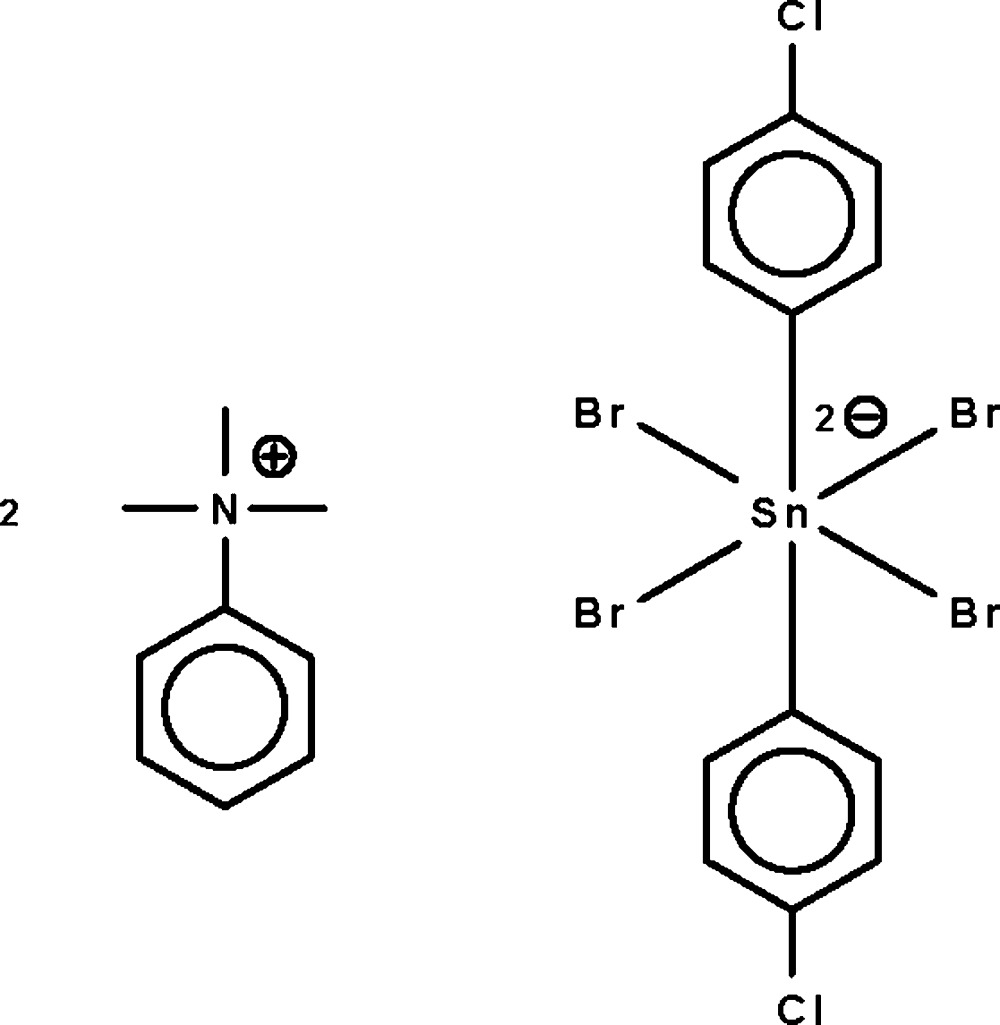


## Experimental

### 

#### Crystal data

(C_9_H_14_N)_2_[SnBr_4_(C_6_H_4_Cl)_2_]
*M* *_r_* = 933.84
Monoclinic, 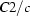

*a* = 25.7930 (3) Å
*b* = 9.0937 (1) Å
*c* = 15.8303 (2) Å
β = 113.4146 (6)°
*V* = 3407.30 (7) Å^3^
*Z* = 4
Mo *K*α radiation
μ = 5.62 mm^−1^
*T* = 293 K
0.30 × 0.25 × 0.20 mm

#### Data collection

Bruker SMART APEX diffractometer
Absorption correction: multi-scan (*SADABS*; Sheldrick, 1996 ▶) *T*_min_ = 0.482, *T*_max_ = 0.756
15816 measured reflections
3916 independent reflections
3427 reflections with *I* > 2σ(*I*)
*R*_int_ = 0.025

#### Refinement

*R*[*F* ^2^ > 2σ(*F* ^2^)] = 0.031
*wR*(*F* ^2^) = 0.098
*S* = 1.24
3916 reflections
181 parameters
H-atom parameters constrained
Δρ_max_ = 1.28 e Å^−3^
Δρ_min_ = −1.03 e Å^−3^

### 

Data collection: *APEX2* (Bruker, 2008 ▶); cell refinement: *SAINT* (Bruker, 2008 ▶); data reduction: *SAINT*; program(s) used to solve structure: *SHELXS97* (Sheldrick, 2008 ▶); program(s) used to refine structure: *SHELXL97* (Sheldrick, 2008 ▶); molecular graphics: *X-SEED* (Barbour, 2001 ▶); software used to prepare material for publication: *publCIF* (Westrip, 2009 ▶).

## Supplementary Material

Crystal structure: contains datablocks global, I. DOI: 10.1107/S160053680904940X/xu2683sup1.cif

Structure factors: contains datablocks I. DOI: 10.1107/S160053680904940X/xu2683Isup2.hkl

Additional supplementary materials:  crystallographic information; 3D view; checkCIF report

## Figures and Tables

**Table 1:** Selected bond lengths (Å)

Sn1—C1	2.156 (3)
Sn1—Br1	2.7368 (4)
Sn1—Br2	2.7386 (4)

**Table 2:** Hydrogen-bond geometry (Å, °)

*D*—H⋯*A*	*D*—H	H⋯*A*	*D*⋯*A*	*D*—H⋯*A*
C15—H15*C*⋯Br1	0.96	2.91	3.839 (5)	163

**Fig. 1.**
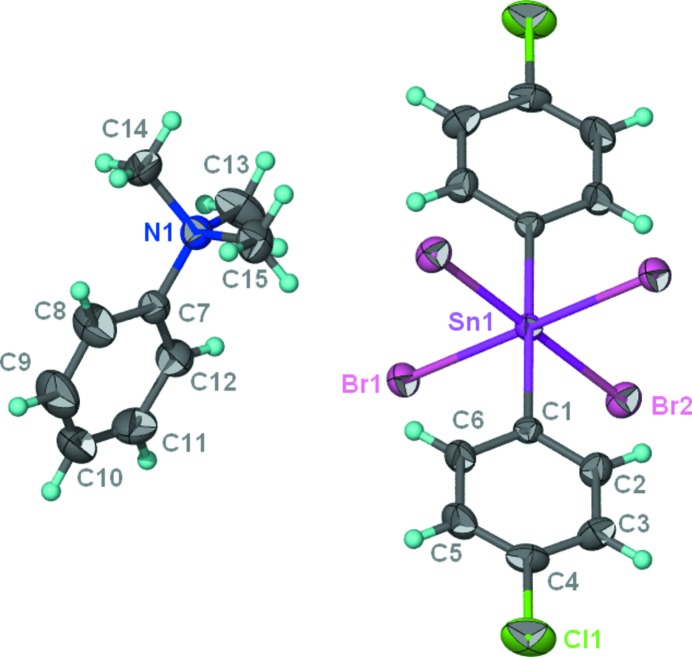
Thermal ellipsoid plot (Barbour, 2001) of the two independent formula units of 2[N(CH3)3(C6H5)] [SnBr4(C6H4Cl)2] at the 50% probability level; hydrogen atoms are drawn as spheres of arbitrary radius.

## supplementary crystallographic information

### Experimental

In an attempt to cleave a tin-carbon bond in a tetraorganotin compound, tetrakis(4-chlorophenyl)tin (0.57 g, 1 mmol) and trimethylphenylammonium tribromide (0.38 g, 1 mmol) were heated in ethanol for six hours. Colorless crystals of the stannate separated from the solution after a few days.

### Refinement

Carbon-bound H-atoms were placed in calculated positions (C–H 0.93–0.96 Å) and were included in the refinement in the riding model approximation, with *U*(H) set to 1.2–1.5*U*(C).

The final difference Fourier map had peak near Sn1 and a hole near Br2.

The refinement program suggested a rather larger second parameter for the weighting scheme. The scheme was arbitrarily selected as (0.05 5.00), which led to a satisfactory, albeit somewhat large, goodness-of-fit.

### Crystal data

**Table:**

(C_9_H_14_N)_2_[SnBr_4_(C_6_H_4_Cl)_2_]	*F*(000) = 1816
*M**_r_* = 933.84	*D*_x_ = 1.820 Mg m^−^^3^
Monoclinic, *C*2/*c*	Mo *K*α radiation, λ = 0.71073 Å
Hall symbol: -C 2yc	Cell parameters from 946 reflections
*a* = 25.7930 (3) Å	θ = 2.4–28.2°
*b* = 9.0937 (1) Å	µ = 5.62 mm^−^^1^
*c* = 15.8303 (2) Å	*T* = 293 K
β = 113.4146 (6)°	Block, colorless
*V* = 3407.30 (7) Å^3^	0.30 × 0.25 × 0.20 mm
*Z* = 4	

### Data collection

**Table:**

Bruker SMART APEX diffractometer	3916 independent reflections
Radiation source: fine-focus sealed tube	3427 reflections with *I* > 2σ(*I*)
graphite	*R*_int_ = 0.025
ω scans	θ_max_ = 27.5°, θ_min_ = 1.7°
Absorption correction: multi-scan (*SADABS*; Sheldrick, 1996)	*h* = −33→33
*T*_min_ = 0.482, *T*_max_ = 0.756	*k* = −11→11
15816 measured reflections	*l* = −20→20

### Refinement

**Table:**

Refinement on *F*^2^	Primary atom site location: structure-invariant direct methods
Least-squares matrix: full	Secondary atom site location: difference Fourier map
*R*[*F*^2^ > 2σ(*F*^2^)] = 0.031	Hydrogen site location: inferred from neighbouring sites
*wR*(*F*^2^) = 0.098	H-atom parameters constrained
*S* = 1.24	*w* = 1/[σ^2^(*F*_o_^2^) + (0.05*P*)^2^ + 5.0*P*] where *P* = (*F*_o_^2^ + 2*F*_c_^2^)/3
3916 reflections	(Δ/σ)_max_ = 0.001
181 parameters	Δρ_max_ = 1.28 e Å^−^^3^
0 restraints	Δρ_min_ = −1.03 e Å^−^^3^

### Fractional atomic coordinates and isotropic or equivalent isotropic displacement parameters (Å^2^)

**Table:**

	*x*	*y*	*z*	*U*_iso_*/*U*_eq_	
Sn1	0.2500	0.7500	0.5000	0.02955 (10)	
Br1	0.183541 (17)	0.69102 (4)	0.59433 (3)	0.04572 (12)	
Br2	0.292712 (17)	0.99167 (4)	0.60875 (3)	0.04584 (12)	
Cl1	0.45173 (6)	0.34442 (18)	0.80689 (10)	0.0818 (4)	
N1	0.13915 (14)	0.2207 (3)	0.5931 (2)	0.0400 (7)	
C1	0.31591 (13)	0.6149 (3)	0.5957 (2)	0.0295 (6)	
C2	0.30386 (17)	0.4780 (4)	0.6223 (3)	0.0422 (8)	
H2	0.2670	0.4426	0.5964	0.051*	
C3	0.34514 (18)	0.3939 (4)	0.6858 (3)	0.0466 (9)	
H3	0.3366	0.3028	0.7037	0.056*	
C4	0.39961 (17)	0.4473 (5)	0.7226 (3)	0.0473 (9)	
C5	0.41310 (16)	0.5804 (5)	0.6971 (3)	0.0518 (10)	
H5	0.4502	0.6141	0.7222	0.062*	
C6	0.37092 (15)	0.6646 (4)	0.6334 (3)	0.0420 (8)	
H6	0.3798	0.7556	0.6158	0.050*	
C7	0.08627 (16)	0.2803 (4)	0.5981 (2)	0.0383 (8)	
C8	0.0346 (2)	0.2255 (6)	0.5397 (4)	0.0658 (13)	
H8	0.0325	0.1501	0.4988	0.079*	
C9	−0.0136 (2)	0.2830 (8)	0.5424 (5)	0.0817 (17)	
H9	−0.0485	0.2469	0.5022	0.098*	
C10	−0.0121 (2)	0.3909 (6)	0.6018 (4)	0.0702 (14)	
H10	−0.0454	0.4290	0.6026	0.084*	
C11	0.0390 (3)	0.4433 (6)	0.6607 (4)	0.0732 (15)	
H11	0.0403	0.5161	0.7027	0.088*	
C12	0.0888 (2)	0.3908 (5)	0.6594 (3)	0.0564 (11)	
H12	0.1233	0.4291	0.6990	0.068*	
C13	0.1911 (2)	0.2531 (7)	0.6789 (3)	0.0683 (14)	
H13A	0.1960	0.3575	0.6867	0.102*	
H13B	0.2236	0.2107	0.6731	0.102*	
H13C	0.1867	0.2115	0.7313	0.102*	
C14	0.1361 (2)	0.0572 (5)	0.5795 (5)	0.0728 (15)	
H14A	0.1059	0.0337	0.5218	0.109*	
H14B	0.1292	0.0111	0.6286	0.109*	
H14C	0.1712	0.0222	0.5795	0.109*	
C15	0.1479 (2)	0.2913 (6)	0.5144 (3)	0.0573 (11)	
H15A	0.1171	0.2660	0.4577	0.086*	
H15B	0.1827	0.2570	0.5129	0.086*	
H15C	0.1494	0.3961	0.5221	0.086*	

### Atomic displacement parameters (Å^2^)

**Table:**

	*U*^11^	*U*^22^	*U*^33^	*U*^12^	*U*^13^	*U*^23^
Sn1	0.02499 (16)	0.03104 (17)	0.03048 (17)	0.00219 (11)	0.00875 (12)	0.00523 (11)
Br1	0.0450 (2)	0.0460 (2)	0.0533 (2)	0.00187 (16)	0.02717 (18)	0.00690 (16)
Br2	0.0481 (2)	0.0417 (2)	0.0424 (2)	−0.00224 (16)	0.01229 (17)	−0.00120 (15)
Cl1	0.0651 (8)	0.0921 (9)	0.0723 (8)	0.0379 (7)	0.0105 (6)	0.0363 (7)
N1	0.0452 (18)	0.0404 (15)	0.0392 (16)	−0.0008 (13)	0.0219 (14)	0.0015 (12)
C1	0.0289 (15)	0.0301 (14)	0.0286 (14)	0.0038 (12)	0.0105 (12)	0.0032 (11)
C2	0.040 (2)	0.0401 (18)	0.0415 (19)	−0.0019 (16)	0.0111 (16)	0.0051 (15)
C3	0.055 (2)	0.0371 (18)	0.043 (2)	0.0073 (17)	0.0149 (18)	0.0098 (15)
C4	0.044 (2)	0.053 (2)	0.0394 (19)	0.0212 (18)	0.0114 (16)	0.0108 (17)
C5	0.0280 (18)	0.062 (3)	0.057 (2)	0.0037 (18)	0.0080 (17)	0.010 (2)
C6	0.0301 (17)	0.0438 (19)	0.048 (2)	0.0015 (15)	0.0110 (15)	0.0100 (16)
C7	0.040 (2)	0.0405 (18)	0.0363 (18)	−0.0006 (15)	0.0168 (16)	0.0021 (14)
C8	0.049 (3)	0.081 (3)	0.058 (3)	−0.008 (2)	0.012 (2)	−0.021 (2)
C9	0.039 (3)	0.102 (4)	0.089 (4)	−0.008 (3)	0.010 (3)	−0.007 (3)
C10	0.053 (3)	0.069 (3)	0.099 (4)	0.012 (2)	0.041 (3)	0.016 (3)
C11	0.083 (4)	0.055 (3)	0.098 (4)	0.009 (3)	0.053 (3)	−0.009 (3)
C12	0.051 (2)	0.051 (2)	0.068 (3)	−0.005 (2)	0.024 (2)	−0.014 (2)
C13	0.047 (3)	0.105 (4)	0.047 (2)	0.016 (3)	0.013 (2)	−0.003 (2)
C14	0.082 (4)	0.040 (2)	0.120 (5)	0.001 (2)	0.065 (4)	−0.001 (3)
C15	0.070 (3)	0.064 (3)	0.051 (2)	−0.006 (2)	0.038 (2)	0.004 (2)

### Geometric parameters (Å, °)

**Table:**

Sn1—C1^i^	2.156 (3)	C7—C12	1.380 (6)
Sn1—C1	2.156 (3)	C7—C8	1.380 (6)
Sn1—Br1	2.7368 (4)	C8—C9	1.365 (8)
Sn1—Br1^i^	2.7368 (4)	C8—H8	0.9300
Sn1—Br2^i^	2.7386 (4)	C9—C10	1.349 (8)
Sn1—Br2	2.7386 (4)	C9—H9	0.9300
Cl1—C4	1.743 (4)	C10—C11	1.362 (8)
N1—C14	1.499 (5)	C10—H10	0.9300
N1—C15	1.495 (5)	C11—C12	1.377 (7)
N1—C7	1.499 (5)	C11—H11	0.9300
N1—C13	1.510 (6)	C12—H12	0.9300
C1—C6	1.379 (5)	C13—H13A	0.9600
C1—C2	1.389 (5)	C13—H13B	0.9600
C2—C3	1.371 (5)	C13—H13C	0.9600
C2—H2	0.9300	C14—H14A	0.9600
C3—C4	1.378 (6)	C14—H14B	0.9600
C3—H3	0.9300	C14—H14C	0.9600
C4—C5	1.364 (6)	C15—H15A	0.9600
C5—C6	1.384 (5)	C15—H15B	0.9600
C5—H5	0.9300	C15—H15C	0.9600
C6—H6	0.9300		
			
C1^i^—Sn1—C1	180.000 (1)	C1—C6—H6	119.7
C1^i^—Sn1—Br1	90.29 (8)	C12—C7—C8	119.8 (4)
C1—Sn1—Br1	89.71 (8)	C12—C7—N1	120.8 (4)
C1^i^—Sn1—Br1^i^	89.71 (8)	C8—C7—N1	119.4 (4)
C1—Sn1—Br1^i^	90.29 (8)	C9—C8—C7	119.4 (5)
Br1—Sn1—Br1^i^	180.0	C9—C8—H8	120.3
C1^i^—Sn1—Br2^i^	90.43 (8)	C7—C8—H8	120.3
C1—Sn1—Br2^i^	89.57 (8)	C8—C9—C10	121.7 (5)
Br1—Sn1—Br2^i^	90.173 (13)	C8—C9—H9	119.1
Br1^i^—Sn1—Br2^i^	89.827 (12)	C10—C9—H9	119.1
C1^i^—Sn1—Br2	89.57 (8)	C11—C10—C9	118.9 (5)
C1—Sn1—Br2	90.43 (8)	C11—C10—H10	120.5
Br1—Sn1—Br2	89.827 (12)	C9—C10—H10	120.5
Br1^i^—Sn1—Br2	90.173 (13)	C10—C11—C12	121.5 (5)
Br2^i^—Sn1—Br2	180.000 (10)	C10—C11—H11	119.2
C14—N1—C15	108.9 (4)	C12—C11—H11	119.2
C14—N1—C7	111.7 (3)	C11—C12—C7	118.6 (5)
C15—N1—C7	109.3 (3)	C11—C12—H12	120.7
C14—N1—C13	107.4 (4)	C7—C12—H12	120.7
C15—N1—C13	107.0 (4)	N1—C13—H13A	109.5
C7—N1—C13	112.5 (3)	N1—C13—H13B	109.5
C6—C1—C2	118.6 (3)	H13A—C13—H13B	109.5
C6—C1—Sn1	120.4 (2)	N1—C13—H13C	109.5
C2—C1—Sn1	121.0 (3)	H13A—C13—H13C	109.5
C3—C2—C1	121.4 (4)	H13B—C13—H13C	109.5
C3—C2—H2	119.3	N1—C14—H14A	109.5
C1—C2—H2	119.3	N1—C14—H14B	109.5
C2—C3—C4	118.6 (4)	H14A—C14—H14B	109.5
C2—C3—H3	120.7	N1—C14—H14C	109.5
C4—C3—H3	120.7	H14A—C14—H14C	109.5
C5—C4—C3	121.6 (3)	H14B—C14—H14C	109.5
C5—C4—Cl1	119.5 (3)	N1—C15—H15A	109.5
C3—C4—Cl1	118.8 (3)	N1—C15—H15B	109.5
C4—C5—C6	119.2 (4)	H15A—C15—H15B	109.5
C4—C5—H5	120.4	N1—C15—H15C	109.5
C6—C5—H5	120.4	H15A—C15—H15C	109.5
C5—C6—C1	120.6 (4)	H15B—C15—H15C	109.5
C5—C6—H6	119.7		
			
Br1—Sn1—C1—C6	−133.2 (3)	C2—C1—C6—C5	−0.7 (6)
Br1^i^—Sn1—C1—C6	46.8 (3)	Sn1—C1—C6—C5	177.9 (3)
Br2^i^—Sn1—C1—C6	136.6 (3)	C14—N1—C7—C12	140.8 (5)
Br2—Sn1—C1—C6	−43.4 (3)	C15—N1—C7—C12	−98.7 (5)
Br1—Sn1—C1—C2	45.3 (3)	C13—N1—C7—C12	20.0 (5)
Br1^i^—Sn1—C1—C2	−134.7 (3)	C14—N1—C7—C8	−40.4 (6)
Br2^i^—Sn1—C1—C2	−44.8 (3)	C15—N1—C7—C8	80.2 (5)
Br2—Sn1—C1—C2	135.2 (3)	C13—N1—C7—C8	−161.2 (4)
C6—C1—C2—C3	1.2 (6)	C12—C7—C8—C9	0.9 (8)
Sn1—C1—C2—C3	−177.4 (3)	N1—C7—C8—C9	−178.0 (5)
C1—C2—C3—C4	−0.8 (6)	C7—C8—C9—C10	−1.0 (10)
C2—C3—C4—C5	−0.2 (6)	C8—C9—C10—C11	−0.1 (10)
C2—C3—C4—Cl1	177.4 (3)	C9—C10—C11—C12	1.4 (9)
C3—C4—C5—C6	0.7 (7)	C10—C11—C12—C7	−1.5 (8)
Cl1—C4—C5—C6	−176.9 (3)	C8—C7—C12—C11	0.3 (7)
C4—C5—C6—C1	−0.2 (7)	N1—C7—C12—C11	179.2 (4)

Symmetry codes: (i) −*x*+1/2, −*y*+3/2, −*z*+1.

### Hydrogen-bond geometry (Å, °)

**Table:**

*D*—H···*A*	*D*—H	H···*A*	*D*···*A*	*D*—H···*A*
C15—H15C···Br1	0.96	2.91	3.839 (5)	163
